# Coping with long-COVID stigma: The role of self-compassion and self-coldness

**DOI:** 10.1177/20551029251349409

**Published:** 2025-07-26

**Authors:** Maor Shani, Kilian Wübbelt

**Affiliations:** 1 Institute for Psychology, 9186 Osnabrück University, Germany; 2 Department of Social Sciences, Ariel University, Israel

**Keywords:** long-COVID, stigma, self-compassion, self-coldness, well-being, flourishing in life

## Abstract

Long COVID has been associated with stigmatization, prompting exploration of coping mechanisms. This cross-sectional study examined whether self-compassion and self-coldness mediate long-COVID stigma’s effects on well-being. We surveyed 201 German adults with long-COVID (89% female; *M*_*age*_ = 43.27, *SD* = 10.57). Most were officially diagnosed (88%), and 93% still experienced long-COVID symptoms at the time of survey. Measures included stigma, self-compassion, self-coldness, subjective well-being (SWB), and flourishing. Long-COVID stigma negatively correlated with SWB and flourishing. Higher self-compassion and lower self-coldness predicted better outcomes. Internalized stigma predicted lower flourishing through decreased self-compassion and increased self-coldness. In contrast, enacted stigma was associated with higher SWB and flourishing through lower self-coldness. Overall, mediation effects via self-coldness were significantly stronger than those via self-compassion, particularly in flourishing. These findings highlight the interplay between stigma, self-relating, and well-being, indicating both adaptive and maladaptive pathways. Interventions promoting self-compassion and reducing self-coldness may support holistic long-COVID care.

The COVID-19 pandemic has led to significant shifts in health, economic, and social domains, with ongoing scientific attention directed toward the enduring effects known as long-COVID. This condition, characterized by persistent multi-organ symptoms, substantially impacts daily functioning (Al-Aly and Topol, 2024; Jiao et al., 2024). The World Health Organization defines long-COVID as occurring in individuals with confirmed or probable prior infection, manifesting symptoms 3 months post-initial infection, lasting for over 2 months, and not attributable to alternative diagnoses (Soriano et al., 2022). Prevalence studies indicate variable rates, ranging from 8.5% to 26.4% in adults (Mandel et al., 2024). The psychosocial ramifications of long-COVID symptoms are profound and significantly impact patients’ physical, occupational, and emotional well-being (Bautista-Rodriguez et al., 2023; Fancourt et al., 2023). Research indicates that long-COVID predicts elevated levels of depression, anxiety, and other psychiatric disorders beyond other predictors (Metelkina-Fernandez et al., 2024; Sirotiak et al., 2023). In Germany, Schröder et al. (2024) observed diminished health-related quality of life in long-COVID patients compared to healthy controls, highlighting the critical need for comprehensive management strategies to support both mental health and enhance recovery (Mandel et al., 2024).

## Stigmatization of long-COVID

Recent studies in health psychology have begun exploring the potential detrimental effects of long-COVID stigmatization, as both the condition and its associated stigma become more prevalent (Clutterbuck et al., 2024; Damant et al., 2023; Pantelic et al., 2022; Samper-Pardo et al., 2023; Scholz et al., 2023). Stigmatization is a complex process involving the intersection of labeling, negative stereotyping, and power asymmetry pertaining to individual or group characteristics (Andersen et al., 2022). Goffman (1986) viewed stigma as profoundly discrediting an individual, emphasizing the discriminative experience of social stigma within social interactions (Pescosolido and Martin, 2015). Health-related stigma is defined as a social process, experienced or anticipated, characterized by exclusion, rejection, or devaluation resulting from an adverse social judgment about a person identified with a particular health problem (Scambler, 2009). Research has demonstrated that stigma often leads to delayed healthcare seeking, reduced treatment adherence, and increased social isolation, all of which can exacerbate health issues and psychological distress (Earnshaw and Quinn, 2012; Stangl et al., 2019), and may seriously compromise the quality of care received (Nyblade et al., 2019). This can create negative healthcare experiences for stigmatized individuals, reinforce their reluctance to seek future care, and perpetuate a cycle of poor health outcomes.

The stigma directed toward individuals with long-COVID has, in many ways, evolved from the widespread stigma observed during the acute phases of COVID-19. Early in the pandemic, fear of contagion and the perceived need to identify “others” as responsible for transmission fueled prejudice, xenophobia, and discrimination (Bhanot et al., 2021; Devakumar et al., 2020; Ransing et al., 2020). These dynamics targeted people suspected of infection, certain ethnicities, and healthcare workers (Chew et al., 2020; He et al., 2020). As the pandemic progressed, long-COVID or post-acute sequelae of SARS-CoV-2 infection began to garner attention, bringing a shift in stigma’s focal points (Crook et al., 2021; Davis et al., 2023). In particular, individuals with persistent COVID-19 symptoms were often blamed for not recovering “fast enough” or were viewed with lingering fears of contagiousness (Ladds et al., 2020). However, the most distinctive element of long-COVID stigma is the delegitimization of ongoing, and often invisible, symptoms. This dismissal is exacerbated by a lack of definitive biomarkers and incomplete scientific consensus, a reality sometimes described as “medical gaslighting” (Au et al., 2022; Plastina, 2025). Moreover, marginalized populations—such as racialized minorities and women—may experience additional stigmas that magnify negative healthcare encounters (Siu et al., 2023). Collectively, these developments mark a transition from fear-based exclusion toward long-term doubts about the legitimacy of chronic COVID-19 sequelae (Pantelic et al., 2022; Sudre et al., 2021). Long-COVID stigma is further influenced by an array of factors, including misinformation about the disease’s prevention and treatment, as well as the invisibility or subjective nature of many long-COVID symptoms (Pantelic et al., 2022; Scholz et al., 2023). Because symptoms are frequently complex and fluctuating, sufferers may be perceived as exaggerating their health issues, contributing to skepticism and invalidation (Connor et al., 2024). Additionally, uncertainty about the pathophysiology of long-COVID can prompt fear among the public, bolstered by concerns that these individuals might still transmit the virus or strain health resources (Clutterbuck et al., 2024; Scholz et al., 2023). This confluence of misinformation, symptom invisibility, and ongoing anxiety around COVID-19 perpetuates the stigmatization of those living with long-COVID, often impeding their access to supportive services or undermining their recovery (Pantelic et al., 2022).

Stigmatization can occur through enacted stigma (direct discrimination), internalized stigma (patients internalizing negative perceptions), and anticipated stigma (patients expecting judgment due to their condition) (Earnshaw et al., 2013; Pantelic et al., 2022). A pioneering survey in the United Kingdom (UK) found that 95% of respondents felt stigmatized at least occasionally, with 76% experiencing it often or always (Pantelic et al., 2022). Moreover, people with long-COVID often feel dismissed when reporting symptoms to healthcare professionals, who may attribute symptoms to mental health issues rather than long-COVID. This can lead to avoidance of care-seeking and internalized stigma, especially among those with intersecting stigmas due to marginalized identities (Clutterbuck et al., 2024). These findings highlight the pervasive nature of stigmatization among long-COVID patients and the need for further research to understand its impact on their well-being and overall quality of life.

## The detrimental effects of stigma on well-being

The concepts of *well-being* and *flourishing* capture various aspects of positive functioning and life satisfaction. Subjective well-being (SWB) refers to individuals’ perceptions of their life experiences, emphasizing emotional states and overall positive functioning (Diener et al., 2010). Flourishing in life, in contrast, pertains to optimal human functioning and achieving one’s full potential, encompassing both hedonic and eudaimonic elements (Huppert and So, 2013; Mesurado et al., 2021; Seligman, 2011). While SWB primarily focuses on an individual’s cognitive and affective evaluations of their life, flourishing encompasses a broader range of psychological and social functioning, including elements such as personal growth, purpose in life, and positive relationships.

Stigma significantly impairs individuals’ health-seeking behaviors and overall quality of life, leading to increased health-related stress, negative self-concepts, social isolation, and greater illness preoccupation (Adom et al., 2021; Hatzenbuehler et al., 2013; O’Donnell and Habenicht, 2022). Consequently, stigma can have a profound impact on various aspects of well-being. Self-determination theory (SDT) provides a framework for understanding how stigma can negatively impact well-being and psychological functioning. SDT posits that humans have an innate predisposition towards growth but are sensitive to their environments (Ryan and Deci, 2017). Stigmatizing experiences can therefore thwart feelings of competence and relatedness (Megías et al., 2018), potentially predisposing individuals to internalize stigma-related messages.

Consistent with these notions, empirical research has demonstrated the negative impact of stigma on SWB in the context of mental illness (Markowitz, 1998; Pérez-Garín et al., 2015). Research has also found that stigma practices compromise ‘human flourishing’ and to constrain life choices (Pemberton, 2016), by limiting individuals’ opportunities and leading to social exclusion. Recent research has documented the negative impact of stigma among long-COVID patients, with elevated levels of stigmatization positively correlated with depression, anxiety, and loneliness, and negatively correlated with perceived social support and quality of life (Damant et al., 2023; Samper-Pardo et al., 2023).

These findings highlight the need for increased awareness and education about long-COVID to create informed environments (Buonsenso et al., 2023) and targeted interventions to mitigate stigma’s negative consequences (Scholz et al., 2023). However, the relationship between long-COVID stigma and psychological flourishing warrants further examination.

## Self-compassion and well-being

Given the pervasive negative outcomes of stigmatization, identifying adaptive coping mechanisms and sources of resilience that may buffer these effects is crucial, also in the case of long-COVID (Scholz et al., 2023). In this regard, self-compassion emerges as a potentially effective coping resource in dealing with long-COVID stigma. Self-compassion encompasses the practice of extending warmth and empathy towards oneself in moments of struggle, acknowledging that challenges and imperfections are inherent aspects of the universal human condition (Neff, 2003a, 2003b). It involves fostering a gentle, supportive attitude towards one’s own experiences of hardship and perceived shortcomings. Self-compassion has been a key element in many cognitive-based therapeutic approaches, characterized by an individual’s capacity to embrace their own difficulties and pain with gentleness and understanding, rather than harsh self-criticism (Neff, 2003a, 2023).

Research has shown that self-compassion is associated with adaptive coping strategies and reduced negative emotions in the face of adversity (Allen and Leary, 2010). Individuals with higher levels of self-compassion exhibit greater psychological flexibility and are more likely to engage in positive health behaviors (Sirois et al., 2015). Studies have demonstrated that individuals with elevated levels of self-compassion tend to experience enhanced psychological well-being, including increased happiness, a more positive outlook, greater contentment with life, and heightened motivation (Hollis-Walker and Colosimo, 2011; Neff et al., 2007). Accordingly, meta-analyses concluded that self-compassion is negatively associated with depression, anxiety, and stress (MacBeth and Gumley, 2012), and positively associated with SWB (Zessin et al., 2015). Self-compassion has also been linked to flourishing, with adaptive sub-components being positively associated with flourishing, while maladaptive sub-components were negatively associated (Chan et al., 2022; Wang et al., 2024).

Regarding the dimensional structure of self-compassion, recent advances have reconceptualized self-compassion as comprising two factors: self-compassion (mindfulness, common humanity, and self-kindness) and self-coldness (isolation, over-identification, and self-judgment) (Brenner et al., 2017). This dual-factor model aligns with the notion of social mentality (Gilbert, 2005), which posits that self-compassion and self-coldness operate through distinct neurobiological systems (Falconer et al., 2015; Gilbert, 2005; Longe et al., 2010). López et al. (2015) and Costa et al. (2016) proposed that self-compassion taps into the mammalian safeness system, while self-coldness taps into the threat defense system. Emerging research suggests that both self-compassion and self-coldness have distinct roles in mental health and well-being (López et al., 2015; Muris and Petrocchi, 2017). Exploring the potentially different mediating mechanisms of self-compassion and self-coldness between long-COVID stigma and well-being can help identify precise intervention targets.

## Self-compassion as a potential mediator in the relationship between long-COVID stigma and well-being

Self-compassion has been explored as a potential adaptive coping practice for various health-related stigmas, including mental illnesses (Heath et al., 2018), autism (Wong et al., 2016), HIV (Brion et al., 2014; Yang and Mak, 2017), obesity (Fekete et al., 2021; Hilbert et al., 2015), and Hepatitis B (Wang et al., 2024). Self-compassionate individuals are more likely to maintain a positive self-view, experience less shame, and engage in adaptive coping strategies when facing stigma (Neff et al., 2007). In the context of long-COVID, where stigma may arise due to the poorly understood nature of the condition (Pantelic et al., 2022), cultivating self-compassion may be particularly beneficial for maintaining SWB and facilitating adaptive coping.

Empirical studies treated self-compassion as both a moderator and a mediator in the relationship between stressors and well-being. The moderating role aligns with the stress-buffering hypothesis (Cohen and Wills, 1985), suggesting that self-compassion can attenuate the negative impact of stigma on mental health (Heath et al., 2018; Mróz, 2022; Wong et al., 2016; Yang and Mak, 2017). However, mediational models may be more appropriate, as they recognize that suffering and self-compassion are interconnected and demonstrate how stigma levels in one’s environment may influence the ability to use self-compassion as an adaptive coping skill. The mediating role of self-compassion can be understood through various theoretical perspectives. Social mentality theory posits that self-compassion activates the self-soothing system, helping regulate negative emotions and promoting feelings of safety (Gilbert, 2005). From the perspective of SDT, self-compassion may support need satisfaction by promoting a more authentic relationship with oneself and maintaining a sense of relatedness even in the face of stigma (Searle et al., 2017). Based on these theories, Stringer and colleagues (2018) developed a model demonstrating how self-compassion may link self-stigmatization and resilience. The development of sustained self-compassion may be a turning point for people dealing with self-stigma, signaling a key perceptual shift that resolves conflicting views of oneself and reduces perceived power imbalances.

However, empirical research has frequently found that self-compassion can sometimes function maladaptively as a coping mechanism. For example, research has found that as individuals experience weight stigma (Fekete et al., 2021) and stigma related to mental illness or HIV (Yang and Mak, 2017), their self-stigma negatively predicts self-compassion. Similarly, Wang et al. (2024) found that self-stigma may reduce self-compassion and increase vulnerability to psychosocial distress among Hepatitis B patients, while high levels of self-compassion can buffer this effect. These findings suggest that people experiencing long-COVID stigma may engage in maladaptive coping through self-coldness, thereby minimizing self-compassion techniques.

## The current study

This cross-sectional study examined the role of self-compassion in coping with long-COVID stress and stigma in a German sample. Despite growing recognition of long-COVID, there is limited research on its psychosocial aspects, particularly concerning stigma, self-compassion, and well-being (Lopez-Leon et al., 2021). Understanding stigmatization and its alleviation is critical due to its impact on well-being dimensions such as resilience, self-efficacy, and community engagement. Accordingly, this study aimed to inform interventions that enhance self-compassion, promoting resilience and psychological flourishing. Following recent research reviewed above (Brenner et al., 2017; Costa et al., 2016; López et al., 2015), we adopted the two-subscale approach to self-compassion distinguishing between self-compassion (or self-warmth) and self-coldness, grasping compassionate versus uncompassionate self-responses, respectively.

**Figure 1.**
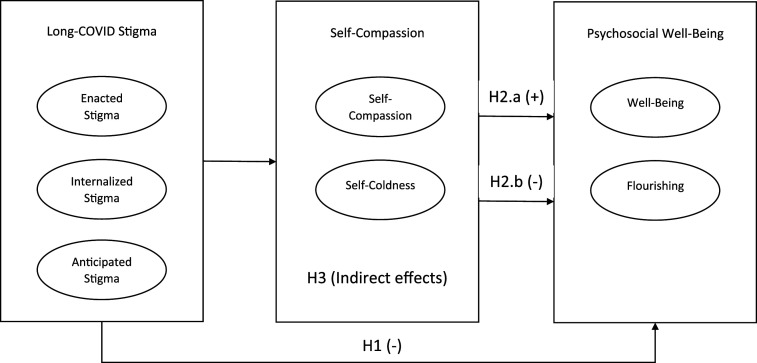
Conceptual model.

The conceptual model of the current research is presented in Figure 1. Examining both SWB and flourishing as well-being outcomes in stigma research provided a more comprehensive understanding of the relationships between stigma and well-being, and enabled us to capture not only the immediate emotional and cognitive effects of stigma (through SWB) but also its broader implications for personal development and social functioning (through flourishing). This may be particularly valuable in studying complex phenomena like stigma, where the effects may manifest in various domains of an individual’s life. Based on our literature review, we hypothesized that among people with long-COVID, enacted, internalized, and anticipated stigma will be negatively associated with SWB and flourishing, such that higher stigma will predict lower SWB and lower psychological flourishing (H1). We expected that self-compassion would positively predict SWB and psychological flourishing (H2.a), while self-coldness would negatively predict these outcomes (H2.b). Considering inconsistencies in theories and prior findings, we expected self-compassion and self-coldness to mediate the relationship between stigma and SWB and flourishing (H3), but did not form concrete hypotheses pertaining to the direction of these relationships. We interpreted our findings based on the direction of the mediation paths. An adaptive coping path was indicated if higher perceived stigma predicted either higher self-compassion or lower self-coldness, which in turn led to higher SWB or flourishing. Conversely, a maladaptive path was suggested if higher perceived stigma predicted either lower self-compassion or higher self-coldness, resulting in lower SWB or flourishing. This latter scenario implied that higher stigma was associated with negative coping mechanisms.

## Method

### Participants and procedure

Participants were German-proficient adults aged 18 and over, recruited through long-COVID Germany-based self-help groups on Facebook, Instagram, and X (formerly Twitter). Long-COVID was defined as symptoms persisting for more than 3 months post-COVID-19 diagnosis without alternative explanations (Seo et al., 2024). The sample included individuals self-identifying as having long-COVID without official diagnosis due to diagnostic challenges (Soriano et al., 2022) and to capture diverse subjective experiences irrespective of diagnostic status (Kingstone et al., 2020; Pantelic et al., 2022).

Participants completed an anonymous 15-min online survey, with optional entry into a 100 Euro raffle. After obtaining informed consent, participants completed self-report measures of demographics, illness-related variables, well-being, self-compassion, and long-COVID stigma, with item randomization within scales to avoid priming effects. *A priori* power analysis using Monte Carlo simulations (Schoemann et al., 2017) determined that 176 participants were needed for significant mediation effects (1 - β = .84, 95% CI [.80, .87]). Of 306 initial respondents, 21 were excluded for inattention (i.e., failing to properly answer an attention-checking question) and 84 for incomplete (<20%) responses, resulting in a final sample of *N* = 201.

The sample predominantly consisted of women (89.1%), with 9.0% men, 1.5% identifying as diverse, and one participant not specifying gender. Ages ranged from 21 to 77 years (*M* = 43.27, *SD* = 10.57). With respect to prior COVID-19 infection, 92.5% had an official clinical diagnosis, 7.0% were diagnosed through an antigen test, and 0.5% were self-diagnosed. A majority (62.2%) reported only one COVID-19 infection, whereas 37.8% had two or more prior infections. Pertaining to their long-COVID status, most participants (92.5%) reported suffering from long-COVID at the time of survey, while the rest experienced it in the past or were uncertain about their status. Official diagnoses were reported by 88.1% of participants, while 9.0% were not officially diagnosed and were not excluded, as explained above.

To validate the long-COVID conditions of our participants, we included a checklist of symptoms based on current guidelines for physicians in Germany (Gogoll et al., 2023). This measure captures any symptom reported after the initial infection, encompassing physical (e.g., fatigue, pain), cognitive (e.g., brain fog, memory loss), respiratory (e.g., dyspnoea, palpitations, cough), gastrointestinal (e.g., diarrhea, stomachache, vomiting), and mental health problems (e.g., depression, anxiety, sleep disturbances). Participants were asked to indicate which long-COVID symptoms they are currently or have previously experienced. As shown in Table 1, the most frequently reported symptoms in our sample were fatigue (97.0%), performance limitations (96.5%), muscle and joint pain (79.1%), sleep disturbances (68.7%), and headaches (63.7%). Common symptoms also included depression (31.3%) and anxiety (21.4%). These symptom distributions align well with previous research from German samples (Gogoll et al., 2023) as well as recent meta-analyses (Luo et al., 2024; Surapaneni et al., 2022). For instance, Luo et al. (2024) conducted a meta-analysis of 212 studies and reported that during the first 3 months, fatigue, PTSD, anxiety, dyspnoea, and depression were the most frequently observed symptoms; similar patterns were observed in subsequent follow-up periods. These findings closely match the symptom profile observed in our sample.

**Table 1.** Sample characteristics (*N* = 201).

Variable	*n*	%/
Age	43.27	10.57
21–30	25	12.8
31–45	95	47.3
46–59	69	35.2
≥60	7	3.5
Missing	5	
Gender		
Male	18	9.0
Female	179	89.5
Other	3	1.5
Missing	1	
Level of education		
Elementary	14	7.0
Secondary	47	23.6
High school	43	21.6
Academic degree	95	47.7
Missing	1	
Income		
Under 1000 Euro	30	14.9
1000–2000 Euro	81	40.9
2000–3000 Euro	56	28.3
3000–5000 Euro	27	13.6
5000 Euro and above	4	2.0
Missing	3	
Clinical diagnosis of COVID-19		
Officially diagnosed	186	92.5
Diagnosed through antigen test	14	7.0
Self-diagnosed	1	0.5
Number of COVID-19 infections		
Once	125	62.2
Twice or modre	76	37.8
Clinical diagnosis of Long-COVID		
No	18	9.0
Yes	177	90.8
Missing	6	
Current Long-COVID status		
Currently suffer from long-COVID	186	92.5
Had long-COVID in the past	6	3.0
Not sure, I but think that currently suffer from long-COVID	8	4.0
Not sure, but I think that I suffered from long-COVID in the past	1	0.5
Common symptoms		
Fatigue	195	97.0
Performance limitation	194	96.5
Muscle and joint pain	159	79.1
Sleep disturbance	138	68.7
Headache	128	63.7
Shortness of breath	91	45.3
General pain	68	33.8
Depressive mood	63	31.3
Olfactory and gustatory disorders	52	25.9
Anxiety	43	21.4
Cough	36	17.9

### Measures

#### Long-COVID stigma

We used the Long-COVID Stigma Scale table S1 (LCSS) developed by Pantelic et al. (2022), specifically designed for long-COVID stigma. The LCSS consists of 13 self-report items covering three dimensions: enacted stigma (discrimination and negative treatment), internalized stigma (self-blame and shame), and anticipated stigma (expectations of future discrimination). Participants rated items on a 5-point frequency scale from 0 (*never*) to 4 (*always*). For example, enacted stigma includes items like, “Because of my illness, some people felt uncomfortable with me”. The initial validation in a UK sample showed good internal consistency reliability with Cronbach’s α values of 0.82, 0.88, and 0.86 for anticipated, enacted, and internalized stigma, respectively (Pantelic et al., 2022). We translated the LCSS into German using a back-translation process. To confirm the dimensional structure in our sample, we evaluated model fit using several indices: normed chi-square (χ2/*df*; ≤3), comparative fit index (CFI; ≥0.90), Tucker–Lewis index (TLI; ≥0.90), and root mean square error of approximation (RMSEA; ≤0.08) (Hu and Bentler, 1999). With two item residual covariates added, the three correlated factors outperformed a single factor model and showed good fit: χ^2^(60) = 98.080, χ^2^/*df* = 1.635, CFI = .956, TLI = .943, RMSEA = .056, 90% CI [.035, .076], SRMR = .048 (see Supplemental Tables S2 and S3 for model comparison and factor loadings, respectively). The reliability was confirmed with Cronbach’s Alpha values of .829, .725, and .710 for enacted, internalized, and anticipated stigma, respectively.

#### Self-compassion and self-coldness

We used the German version of the Self-Compassion Scale (SCS-D) by Hupfeld and Ruffieux (2011), originally developed by Neff (2003b). The SCS is a 26-item measure (25 items in our study due to a technical error in one item in the self-judgment scale), evaluating six subscales: self-kindness, self-judgment, common humanity, isolation, mindfulness, and over-identification. Items were rated on a 5-point Likert scale, ranging from 1 (*almost never*) to 5 (*almost always*). Cronbach’s alpha values for the six subscales ranged from .661 for common humanity (4 items) to .870 for self-kindness (5 items), similar to previous validations (Hupfeld and Ruffieux, 2011). Following recent recommendations (Brenner et al., 2017; Costa et al., 2016; López et al., 2015), we divided the subscales into two dimensions: self-compassion (self-kindness, common humanity, mindfulness) and self-coldness (self-judgment, isolation, over-identification). To validate the choice of dimensional structure, we compared four models: single-factor, six correlated factors, two correlated factors, and a second-order latent model (see Supplemental Table S3). Although recent recommendations suggest that a bifactor model may provide a more theoretically valid structure (Neff and Tóth-Király, 2022), this model did not converge in our case. The CFA results indicate that the two-factor conceptualization outperformed the single-factor and original six-factor models, consistent with recent studies (Brenner et al., 2017; Coroiu et al., 2018; Lu et al., 2022) and aligning with Neff’s (2023) recommendation for higher-order modeling. Accordingly, self-compassion was examined as two factors: self-compassion (α = .819 for subscales, α = .888 for items) and self-coldness (α = .878 for subscales, α = .907 for items). The self-compassion subscale assesses empathy and understanding towards oneself, while the self-coldness subscale assesses isolation, amplification of suffering, and self-judgment. Separate scores were calculated, with higher scores indicating higher levels of self-compassion or self-coldness.

#### Subjective well-being

We used the WHO Well-Being Index (WHO-5), a unidimensional instrument measuring positive well-being over the past 2 weeks (Topp et al., 2015). This self-administered questionnaire comprises five positively worded items rated on a 5-point Likert scale, ranging from 0 (*at no time*) to 4 (*all of the time*). Items include, for example: “I have felt active and vigorous” and “I have felt cheerful and in good spirits”. We used the German version validated by Brähler et al. (2007), widely used in German-speaking populations. The WHO-5 has demonstrated excellent psychometric properties, including high internal consistency (Cronbach’s α: 0.84-0.95) and convergent validity (Topp et al., 2015). It has been extensively used in chronic illness research, including long-COVID studies (e.g., Duwel et al., 2023), and in investigations of self-compassion in German-speaking populations (e.g., Dreisoerner et al., 2021). A CFA model showed a satisfactory single-factor model fit (see Supplemental Table S4), confirming that the five items measure together the latent construct of SWB (α = .806).

#### Flourishing

We used the German version of the Flourishing Scale (FS-D) (Esch et al., 2013), based on Diener et al.’s (2010) original scale. The FS is an 8-item self-report measure assessing aspects of eudaimonic well-being and positive functioning. Participants rated items on a 7-point Likert scale ranging from 0 (*completely disagree*) to 6 (*completely agree*), with higher scores indicating higher levels of flourishing. An example item: “I lead a purposeful and meaningful life”. Recent self-compassion research (Chan et al., 2022; Mróz, 2022) has employed the FS, showing high reliability and significant correlations in expected directions. In our sample, the FS-D demonstrated good psychometric properties, with α = .874. CFA validated the single latent variable structure, including covariances between items 3 and 5, resulting in a satisfactory model fit (see Supplemental Table S5).

#### Long-COVID burden

To control for the stress caused by the illness itself, we included a brief measure of subjective long-COVID severity using a single item. Participants were asked to rate the severity of their long-COVID symptoms on a 10-point Likert scale. The item was presented as follows: “Please rate the severity of your long-COVID on a scale from 0 to 10, where 0 indicates that your symptoms are not severe and 10 indicates that your long-COVID symptoms are very severe (0 = *Not severe* … 10 = *Very severe*)”.

#### Demographics and health-related variables

We measured gender, age (in years), income (5-point scale representing income ranges from below 1000 Euros to above 5000 Euros), and duration of illness (time since first experiencing long-COVID symptoms, converted to percentiles to avoid inflated variance). These variables were used as covariates in the model to strengthen the robustness of the findings.

## Results

The prevalence of people experiencing overall stigma *often* or *always* was 28.4% for enacted stigma, 64.2% for internalized stigma, 65.2% for anticipated stigma, and 82.6% overall (see Supplemental Table S6 and Figure S1 in the supplementary file for additional item frequencies). These percentages are slightly higher than those found in a previous UK-based sample (Pantelic et al., 2022). The highest average frequency was observed for anticipated stigma (*M* = 1.908, *SD* = 0.725), followed by internalized stigma (*M* = 1.580, *SD* = 0.831), and enacted stigma (*M* = 1.198, *SD* = 0.793). The differences in means across the three subscales were statistically significant, *F*(2,398) = 95.481, *p* < .001, partial η^2^ = .324, with all pairwise comparisons showing significant differences (*p*s < .001).

Table 2 displays the mean values, standard deviations, and correlation patterns of the variables measured. Significant positive correlations were observed among the three types of stigma. Enacted stigma was strongly associated with internalized stigma and anticipated stigma. Internalized and anticipated stigma were also significantly correlated. These findings suggest that individuals who experience one form of stigma are likely to experience the other forms as well. All three types of stigma showed significant negative correlations with SWB and flourishing. Self-compassion and self-coldness were negatively correlated. Self-compassion showed significant positive correlations with SWB and flourishing, whereas self-coldness had significant negative correlations with these outcomes.

**Table 2.** Means, standard deviations, and bivariate Pearson correlations (r) for the study’s main variables (*N* = 201).

Variable	*M*	*SD*	1	2	3	4	5	6	7
1. Enacted stigma	1.196	0.792							
2. Internalized stigma	1.577	0.830	.512***						
3. Anticipated stigma	1.907	0.725	.658***	.561***					
4. Self-compassion	2.414	0.740	−.131	−.362***	−.212**				
5. Self-coldness	1.575	0.877	.221**	.568***	.381***	−.634***			
6. Subjective well-being	1.599	0.928	−.305***	−.393***	−.295***	.366***	−.463***		
7. Flourishing	4.056	1.067	−.380***	−.404***	−.349***	.463***	−.548***	.555***	

***p < .001. **p < .01. *p < .05.

As expected, self-compassion showed significant positive correlations with SWB and flourishing, suggesting that higher self-compassion is associated with better SWB and flourishing. Conversely, self-coldness had significant negative correlations with SWB and flourishing, indicating that higher self-coldness is linked to poorer SWB and flourishing. Regarding the relationship between stigma and self-compassion, the results showed significant associations. Enacted, internalized, and anticipated stigma domains were all positively correlated with self-coldness, while only internalized and anticipated stigma were negatively and moderately correlated with self-compassion.

Regarding demographics and health-related characteristics, long-COVID burden was weakly associated with enacted stigma (*r* = .197) and SWB (*r* = −.224, *p*s < .01). Length of illness had weak but significant correlations with enacted stigma (*r* = .162) and SWB (*r* = .164, *p*s < .05). Age showed a weak negative correlation with internalized stigma (*r* = −.168), and income was slightly negatively correlated with enacted stigma (*r* = −.163, *p*s < .05).

### Structural equation modeling and path analysis

We initially employed structural equation modeling (SEM) using Mplus version 8.0 (Muthen and Muthen, 2017) to examine the mediating effects of self-compassion and self-coldness on the relationship between long-COVID stigma and SWB. Given the non-normal distribution of some measures (see Supplemental Table S7), we used the Robust Maximum Likelihood (MLR) estimator (Finch and Bolin, 2017). Missing data (<6% per variable) were handled using Full Information Maximum Likelihood (FIML), Little’s MAR test: χ^2^(76) = 90.137, *p* = .128 (Enders and Bandalos, 2001). The initial measurement model, comprising seven latent factors, showed inadequate fit, χ^2^(443) = 807.449, *p* < .001, RMSEA = .064, 95% CI [.057, .071], CFI = .877, TLI = .863, SRMR = .074. After modifications, fit improved to acceptable levels, χ^2^(438) = 737.406, *p* < .001, RMSEA = .058, 95% CI [.051, .066], CFI = .905, TLI = .892, SRMR = .065.

However, we encountered obstacles in achieving model identification, likely due to our sample size being insufficient to estimate 49 parameters. SEM typically requires larger samples, with recommendations suggesting at least ten times the number of estimated parameters for a robust model (Kline and Little, 2023; Wolf et al., 2013). Additionally, the model’s complexity and potential lack of sufficient variation in some variables may have contributed to SEM estimation failure. Given these convergence issues and the complexity of our model, we opted for path analysis, a more parsimonious and robust approach less prone to identification problems (Streiner, 2005), with the same model specification detailed above. Path analysis allows for the estimation of direct and indirect effects, even with non-normal data and small sample sizes (Hayes, 2013). This approach enabled us to maintain the theoretical structure of the model while ensuring its suitability for the available sample size. It also provided a clearer interpretation of the results, which was particularly useful given the complexity of our model.

**Figure 2.**
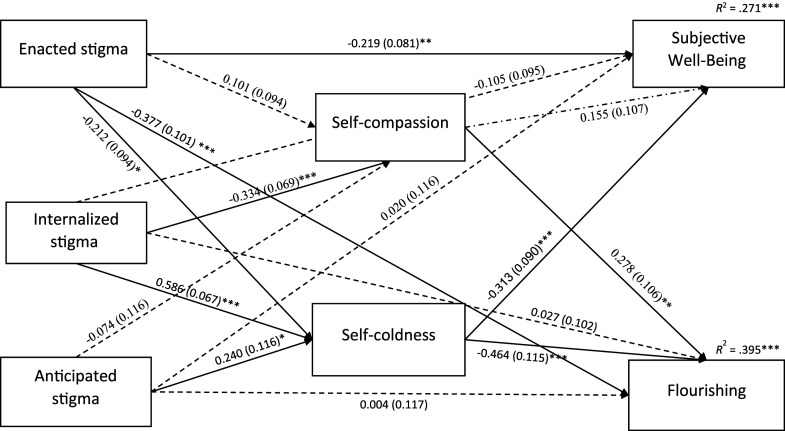
Diagram of path model predicting flourishing and subjective well-being. *Note*. *N* = 201. Path coefficients (*B*s) and standard errors (*SE*s, in parentheses) are unstandardized estimates. Dashed lines represent nonsignificant effects. Covariances among predictors, mediators, and outcomes are included in the model but are not shown graphically. *p < .05, **p < .01, ***p < .001.

The specified path model with coefficients is depicted in Figure 2. Initially, Variance Inflation Factors (VIFs) were examined and found to be lower than 3 in all regressions. Interestingly, in a model including all three stigma domains, enacted stigma significantly predicted lower self-coldness (although the bivariate correlation between enacted stigma and self-coldness was positive), while internalized and anticipated stigma positively predicted self-coldness. Moreover, self-compassion was negatively predicted only by internalized stigma. Self-coldness significantly predicted lower SWB and flourishing, while self-compassion predicted flourishing only.

Subsequently, we examined the mediating role of self-compassion and self-coldness using bootstrapping with 5000 resamples. This method is considered robust for small samples (Hayes, 2013). Specific indirect effects are presented in Table 3. Self-compassion significantly mediated only the relationship between internalized stigma and flourishing, indicating a maladaptive role. The indirect effects through self-compassion for enacted and anticipated stigma were not significant. In contrast, self-coldness significantly mediated the relationships between all three stigma dimensions and both well-being constructs. Specifically, the results demonstrate an adaptive path for enacted stigma, but maladaptive paths for internalized and anticipated stigma. Additionally, direct negative effects remained between enacted stigma and both well-being outcomes, suggesting that other mechanisms may further explain the relationship between long-COVID stigma and well-being.

**Table 3.** Specific indirect effects in the path analysis for the mediation role of self-compassion and self-coldness in the relationship between long-COVID stigma and well-being.

Path of indirect effect	*B*	*SE*	95% bootstrapped *CI*	Adaptive/maladaptive role
Self-compassion				
Enacted stigma → SWB	0.016	0.021	[−0.007, 0.092]	
Internalized stigma → SWB	−0.052	0.038	[−0.129, 0.015]	
Anticipated stigma → SWB	−0.012	0.023	[−0.089, 0.016]	
Enacted stigma → flourishing	0.028	0.030	[−0.017,0.109]	
Internalized stigma → flourishing	−0.093	0.044	[−0.195, −0024]	Maladaptive
Anticipated stigma → flourishing	−0.021	0.034	[−0.100, 0.038]	
Self-coldness				
Enacted stigma → SWB	0.066	0.038	[0.009. 0.158]	Adaptive
Internalized stigma → SWB	−0.184	0.059	[−0.311, −0.077]	Maladaptive
Anticipated stigma → SWB	−0.075	0.045	[−0.184, −0.002]	Maladaptive
Enacted stigma → flourishing	0.098	0.050	[0.016, 0.217]	Adaptive
Internalized stigma → flourishing	−0.272	0.078	[−0.441, −0.131]	Maladaptive
Anticipated stigma → flourishing	−0.111	0.061	[−0.258, −0.007]	Maladaptive

*Note. N* = 201.

SWB: subjective well-being (WHO-5).

To explicitly compare the relative strength of these mediation effects, we computed the total indirect effects via self-compassion (i.e., the sum of the specific indirect effects through self-compassion) and via self-coldness, and then contrasted these effects. For SWB, the total indirect effect via self-compassion was not significant (*B* = −0.048, *SE* = 0.038, 95% CI [-0.135, 0.021], *p* = .204), whereas the total indirect effect via self-coldness was significant (*B* = −0.192, *SE* = 0.062, 95% CI [-0.341, −0.088], *p* = .002). The contrast between these mediation paths (self-coldness minus self-compassion) approached significance (*B* = −0.145, *SE* = 0.085, 95% CI [-0.340, −0.002], *p* = .088), suggesting that self-coldness tends to mediate the relationship between stigma and SWB more strongly than self-compassion. For flourishing, although both mediation paths were significant—via self-compassion (*B* = −0.085, *SE* = 0.040, 95% CI [−0.183, −0.022], *p* = .033) and via self-coldness (*B* = −0.285, *SE* = 0.079, 95% CI [−0.453, −0.145], *p* < .001)—the mediation effect via self-coldness was significantly stronger than that via self-compassion, as indicated by the significant contrast (*B* = −0.199, *SE* = 0.097, 95% CI [−0.408, −0.026], *p* = .041).

Finally, a robustness analysis that included covariates (gender, age, income, long-COVID burden, and time since symptoms started) confirmed that all significant indirect effects in the original model remained significant (see Supplemental Table S8), thereby providing additional validity to the results.

## Discussion

This study addressed notable research gaps in the emerging literature on long-COVID stigma by examining the role of self-compassion in mitigating its psychosocial impact in a German sample of patients. Overall, the main contribution of this study lies in its novel examination of the relationships between different forms of stigma, self-compassion, self-coldness, and well-being in the context of long-COVID. By investigating self-compassion as a dual-factor construct with differential influence of self-compassion or self-warmth versus self-coldness on well-being (Brenner et al., 2017; Coroiu et al., 2018; Costa et al., 2016; López et al., 2015; Lu et al., 2022; Muris and Petrocchi, 2017), this study provided unique insights into how stigma and self-relating influence mental health in long-COVID patients, extending previous research on self-compassion in health-related stigma (Brion et al., 2014; Heath et al., 2018; Sirois et al., 2015; Wang et al., 2024; Wong et al., 2016).

Consistent with findings from the UK (Pantelic et al., 2022), anticipated stigma was most prevalent, followed by internalized and enacted stigma, though overall prevalence was slightly higher in our German sample compared to the UK sample. Interestingly, illness burden and duration were weakly but significantly related to higher enacted stigma levels, aligning with recent research linking long-COVID stigma to greater symptom burden and impairment (Damant et al., 2023). This relationship suggests that symptoms may play a crucial role in stigmatization processes. Our results further revealed significant negative associations between all three stigma dimensions and both SWB and flourishing, corroborating our hypotheses and contributing to growing evidence of long-COVID’s pervasive and multifaceted stigma (Clutterbuck et al., 2024; Pantelic et al., 2022; Samper-Pardo et al., 2023; Scholz et al., 2023). These findings demonstrate how stigma exacerbates challenges faced by long-COVID patients, who encounter it in social environments, media, and healthcare settings. In addition to its potential negative impact on stress and mental health, internalized or anticipated stigma can lead to delayed healthcare seeking, reduced treatment adherence, increased isolation, and psychological distress (Earnshaw and Quinn, 2012; Stangl et al., 2019). Healthcare stigma can compromise care quality due to provider biases or lack of understanding (Nyblade et al., 2019). Long-COVID stigma may be particularly detrimental to flourishing when intersecting with other forms of social harm. This intersection potentially exacerbates existing inequalities (Clutterbuck et al., 2024; Pemberton, 2016).

As expected, self-compassion positively correlated with well-being indicators, while self-coldness negatively correlated, consistent with literature on self-compassion’s positive outcomes (Hollis-Walker and Colosimo, 2011; Neff, 2023; Neff et al., 2007; Zessin et al., 2015). In addition, path analysis revealed that both self-compassion and self-coldness mediated the relationship between long-COVID stigma and both SWB and flourishing. These findings support self-compassion’s role in psychological health when coping with stigma-related stress, aligning with previous research on self-compassion’s association with resilience and well-being, and add to the growing evidence that self-compassion helps individuals cope with health-related stigma across various populations (Brion et al., 2014; Fekete et al., 2021; Heath et al., 2018; Hilbert et al., 2015; Wang et al., 2024; Wong et al., 2016; Yang and Mak, 2017). These findings align with social mentality theory (Gilbert, 2005), Self-Determination Theory (Ryan and Deci, 2017), and Stringer’s (2018) model. They provide further support for recent research demonstrating that self-compassion serves as an adaptive emotional regulation strategy, promoting well-being, need satisfaction, and resilience in the face of self-stigma.

Two noteworthy findings emerged from our analysis of indirect effects. First, the maladaptive indirect effects of internalized stigma on flourishing through self-compassion, and of internalized and anticipated stigma on well-being and flourishing through self-coldness, align with previous research showing that the mediating role of self-compassion explains rather than counters the negative relationship of stigma with well-being (Fekete et al., 2021; Wang et al., 2024; Yang and Mak, 2017). This is also consistent with research showing that stigma-based burden is associated with lower self-compassion and higher self-coldness, potentially leading to maladaptive coping mechanisms (Jones and Corrigan, 2014). Stigma experiences can significantly undermine an individual’s sense of competence and their ability to form meaningful connections with others (Megías et al., 2018). This erosion of fundamental psychological needs may create a vulnerability that makes individuals more susceptible to internalizing stigmatizing messages and beliefs about themselves (Wang et al., 2024).

Within this framework, self-coldness – a harsh and critical self-attitude – is theorized to operate through the threat defense system (Falconer et al., 2015; Gilbert, 2005). This system is activated when an individual perceives threats to their social status, acceptance, or well-being. In the context of stigma, internalized negative stereotypes and the constant anticipation of discrimination serve as persistent threats. These threats can activate this system, leading to heightened self-criticism, social withdrawal, and other maladaptive coping mechanisms. Over time, chronic activation of the threat defense system and the resulting self-coldness can contribute to the development and exacerbation of various mental health problems. Furthermore, public stigmas may worsen mood and emotion regulation, reducing psychological resources and impeding the development of adequate coping capacities (Lu et al., 2022). Depression, anxiety, and fatigue can negatively impact the ability to be self-compassionate (Pauley and McPherson, 2010), and self-compassion might be challenging to achieve under high perceived stigma (Ledesma, 2014). This maladaptive mechanism, however, suggests potential remedies. Interventions aimed at increasing self-compassionate responses could protect from or alleviate stigma’s negative outcomes.

Second, the significant adaptive indirect effect of enacted stigma on SWB and flourishing through self-coldness is an intriguing finding that contrasts with the maladaptive indirect effects for internalized and anticipated stigma. These results also seemingly contrast the directionality of the zero-order negative correlation: while the bivariate correlation between enacted stigma and self-coldness was positive, the path coefficient was negative when controlling for internalized and anticipated stigma. This discrepancy likely stemmed from suppression effects (Pandey and Elliott, 2010). In this case, internalized and anticipated stigma may have suppressed the true relationship between enacted stigma and self-coldness. This finding underscores the complex interplay between different forms of stigma and their effects on self-relating and well-being outcomes, and emphasizes the importance of examining different dimensions of stigma in a single model. Future research should aim to replicate this finding and further investigate potential suppression effects among different types of stigma in the context of long-COVID. And still, several theoretical explanations may account for these contrasting indirect effects. Enacted stigma, being more overt and external, may trigger active coping strategies that reduce self-coldness, such as seeking social support and validation (Breet et al., 2014). Given that our sample comprised mostly members of a self-help online community, engaging with others who have faced similar discrimination may provide a sense of belonging and understanding (Crabtree et al., 2010). Enacted stigma could also lead to increased self-advocacy and assertiveness, promoting a sense of empowerment and self-worth (Corrigan and Watson, 2002). In contrast, internalized and anticipated stigma, being more insidious, may lead to increased self-criticism and self-coldness (Pérez-Garín et al., 2015; Yang and Mak, 2017). This can potentially result in detrimental effects on SWB and overall functioning (Livingston and Boyd, 2010).

Finally, our findings support the dual-factor model of self-compassion and the necessity of differentiating between self-compassion and self-coldness. While self-compassion appears relevant only in the context of internalized stigma and flourishing, self-coldness consistently mediated the relationships between all three stigma dimensions and both well-being constructs. Comparisons of total effects provided further empirical support for the notion that self-coldness serves as a more robust mediator linking long-COVID stigma to well-being outcomes than self-compassion. These results align with studies linking negative indicators more strongly to mental health problems than positive indicators (Brenner et al., 2017; Muris and Petrocchi, 2017). They also support research highlighting self-coldness as a stronger mediator between stigma experiences and psychological distress (Lu et al., 2022; Watson-Singleton et al., 2019) and well-being (Brenner et al., 2017; Charzyńska et al., 2020). However, they contrast with studies suggesting self-compassion is more strongly associated with positive outcomes (López et al., 2015; Muris and Petrocchi, 2017). Overall, these differential effects underscore the importance of considering both self-compassion and self-coldness within a single model and examining multiple dimensions of stigma, including both perceived and experienced forms.

### Limitations and future directions

This study has several limitations that warrant consideration. First, the cross-sectional design precludes causal inferences regarding the relationships between long-COVID stigma, self-compassion, self-coldness, and well-being. Longitudinal studies are crucial to establish temporal sequences and rule out alternative explanations, such as lower SWB contributing to increased stigma vulnerability (Damant et al., 2023). Such research could also illuminate the evolution of long-COVID stigma and how medical advances influence patient resilience over time (Al-Aly and Topol, 2024). Experimental designs, like self-compassion interventions, could further clarify directional associations, distinguishing these effects from potential confounds (Neff, 2023; Neff et al., 2007).

Second, our recruitment from online support groups may limit the representativeness of the broader long-COVID patient population. Future studies should employ more diverse recruitment methods to obtain more representative samples. The sample’s gender imbalance necessitates future gender-balanced studies to investigate potential differences in long-COVID stigma experiences (Samper-Pardo et al., 2023).

Third, the focus on a German sample may limit generalizability to other cultural contexts. Cross-cultural research is needed to determine the generalizability of findings, as stigma experiences and coping mechanisms can vary with societal norms (Fung et al., 2021), and culture-specific factors may shape the relationships between stigma, self-compassion, and well-being. Moreover, this study examined self-compassion and self-coldness only as mediators, whereas previous research suggested that self-compassion may also function as a moderator in these relationships (e.g., Heath et al., 2018; Mróz, 2022; Yang and Mak, 2017). Future studies should clarify self-compassion’s role by examining its potential moderating and mediating effects.

Finally, the relatively small sample size likely caused convergence issues in the initial SEM analysis. Larger, more diverse samples would enable full utilization of SEM’s advantages and provide a more accurate representation of relationships by accounting for measurement error (Kline and Little, 2023). This would enhance generalizability and allow examination of potential moderators, such as demographic factors or illness characteristics.

### Practical implications

This study’s findings, although preliminary and requiring further support, have potentially important implications for supporting individuals with long-COVID and mitigating the negative effects of stigma on well-being. As long-COVID prevalence continues to increase globally (Ellis, 2024), promoting self-compassion among patients may help them cope adaptively with stigma-related stress and enhance their well-being (Buonsenso et al., 2023; Scholz et al., 2023). Patients who internalize and anticipate stigma may be more likely to engage in maladaptive coping mechanisms, characterized by a deficit in self-compassion and increased self-coldness. To shift from maladaptive to adaptive coping, it is essential to raise awareness about self-compassion’s benefits and provide patients with training and resources to develop this skill. In this regard, our results add to recent studies suggesting meditation and mindfulness as potential adjunctive therapies for addressing underlying pathophysiology (Porter and Jason, 2022) and improving sleep quality and anxiety (Vandenbogaart et al., 2024) in long-COVID patients.

Specifically, healthcare providers and support organizations can empower long-COVID patients by equipping them with self-compassion techniques. Interventions such as mindfulness-based stress reduction (Kabat-Zinn, 2003) and compassion-focused therapy (Gilbert, 2009) can help patients develop a more balanced and accepting relationship with themselves. Compassionate Mind Training (Gilbert and Procter, 2006), for example, can cultivate self-compassion and overall well-being. Similarly, SDT-based interventions can also mitigate the harmful effects of stigma and promote well-being (Ryan and Deci, 2017). Moreover, the high prevalence of long-COVID stigma underscores the need for public health campaigns to reduce stigma and promote accurate information about the condition. Healthcare providers should create inclusive, non-judgmental environments (Buonsenso et al., 2023). Finally, educational interventions should include mass communication campaigns to correct misperceptions about long-COVID and address misconceptions contributing to stigma (Motta et al., 2025).

## Supplemental Material

Supplemental Material - Coping with long-COVID stigma: The role of self-compassion and self-coldness Supplemental Material for Coping with long-COVID stigma: The role of self-compassion and self-coldness by Maor Shani and Kilian Wübbelt in Health Psychology Open

## Data Availability

All data and analysis codes with appropriate outputs are available in an OSF repository under: https://osf.io/4frc6/?view_only=78e5bb4b55eb48dab7b02f92d8461b10.
