# Supplementary material for: Coping with long-COVID stigma: The role of self-compassion and self-coldness
Source: Health Psychol Open. 2025 Jul 26;12:20551029251349409. doi: 10.1177/20551029251349409 (PMC12311885; doi:10.1177/20551029251349409)
Supplement: Supplemental Material - Coping with long-COVID stigma: The role of self-compassion and self-coldness [file sj-pdf-1-hpo-10.1177_20551029251349409.pdf]

Coping with Long-COVID Stigma: The Role of Self-Compassion and Self-Coldness

Online Supplementary Materials

Table S1

Summary of CFAs' Model Fit Indices and Model Comparisons the Long-COVID Stigma Scale

Model specification	χ^2(df)	SCF	$\Delta\chi2$ (df)	AIC	RMSEA [90% CI]	CFI	TLI	SRMR	Modifications
Model 1: One factor model	244.050 (65)***	1.072		6894.678	.117 [.102, .133]	.795	.754	.073	
Model 2A: Three factor model	135.062 (62)***	1.067	Model 2 vs mode 1: 99.982 (3)***	6783.307	.077 [.059, .094]	.916	.895	.068	
Model 2B: Three-factor model (MODIFIED)	98.080 (60)**	1.085	Model 2B vs model 2A: 71.522 (2)***	6749.547	.056 [.035, .076]	.956	.943	.048	With two item residual correlations (items 6-7 and 10-11)

Note. Models estimated with robust standard errors (MLR). CFA = Confirmatory factor analysis; SCF = Scaling correction factor for MLR; CFI = Comparative fit index; CI = Confidence interval; df = Degrees of freedom; RMSEA = Root mean square approximation; SRMR = Standard root means square residual; TLI = Tucker-Lewis Index.
* $p < .05$, ** $p < .01$, *** $p < .001$

Table S2

Standardized Factor loadings of individual stigma items on subscales of internalized, enacted and anticipated stigma using confirmatory factor analysis (N = 201)

Item	Enacted	Internalised	Anticipated
Because of my illness, some people seemed uncomfortable with me	.642		
Because of my illness, some people were unkind to me	.743		
People I care about stopped contacting me after learning I have Long Covid	.603		
People have acted as if I am dishonest since I have had Long Covid	.721		
I have been treated with less respect than other people are because of Long Covid	.791		
I have felt embarrassed about my illness		.638	
I have felt embarrassed because of my physical limitations		.658	
I feel that I have been tainted by Long Covid and am of less value than others because of it		.705	
I have felt like I am very different from other people on account of Long Covid		.368	
Many people tend to think Long Covid isn't a real illness			.567

I feel that some people assume that having Long Covid is a sign of personal weakness			.619
I worry that people with Long Covid lose their jobs when their employers find out			.411
I worry that people may judge me negatively when they learn I have Long Covid			.712
CFI		0.956	
TLI		0.943	
RMSEA		0.056	
SRMR		0.048	
$\chi 2$/df		1.635	
Cronbach's alpha	0.83	0.73	0.71

Note. Models estimated with robust standard errors (MLR). CFA = Confirmatory factor analysis; SCF = Scaling correction factor for MLR; CFI = Comparative fit index; CI = Confidence interval; df = Degrees of freedom; RMSEA = Root mean square approximation; SRMR = Standard root means square residual; TLI = Tucker-Lewis Index.
* p < .05, ** p < .01, *** p < .001

Table S3

Summary of CFAs' Model Fit Indices and Model Comparisons the Self-Compassion Scale

Model specification	χ^2(df)	SCF	$\Delta\chi 2$ (df)	AIC	RMSEA [90% CI]	CFI	TLI	SRMR	Modifications
Model 1: All 25 items loaded on a single underlying factor	807.860 (275)***	1.129		14138.309	.098 [.090, .106]	.755	.733	.084	
Model 2A: Dual factors: Self-compassion (13 items) and self-coldness (12 items) (correlated)	642.165 (274)***	1.129	Model 1 vs. 2A: 165.695 (1) ***	13953.124	.082 [.074, .090]	.831	.815	.075	
Model 2B: Dual factors: Self-compassion (13 items) and self-coldness (12 items) (correlated) - MODIFIED	534.405 (270)***	1.124	Model 2B vs. 2A: 84.785 (4)***	13836.690	.070 [.061, -.078]	.878	.865	.068	4 item residual correlations (items 5-12, 7-10, 13-18, 4-18)
Model 3: Theoretical six factors (correlated)	583.990 (260)***	1.117		13858.074	.073 [.064, .082]	.872	.852	.072	
Model 4:	592.627	1.116	Model	13901.320	.078	.851	.833	.077	

Second-order two-factor model	(268)***		4 vs. Model 3: 8.901 (8)		[.069, .086]				
Model 5A: Item-parceling (two factor model)	67.041 (8)***	1.032		2527.987	.192 [.151, .236]	.910	.831	.045	
Model 5B: Item-parceling (two factor model) MODIFIED	32.286 (6)***	1.077	Model 5A vs. 5B: 38.366 (2)***	2497.570	.148 [.100, .200]	.960	.900	.040	Two item residuals correlations (between common humanity and mindfulness; between isolation and over-identification)

Note. Models estimated with robust standard errors (MLR). CFA = Confirmatory factor analysis; SCF = Scaling correction factor for MLR; CFI = Comparative fit index; CI = Confidence interval; df = Degrees of freedom; RMSEA = Root mean square approximation; SRMR = Standard root means square residual; TLI = Tucker-Lewis Index.
* p < .05, ** p < .01, *** p < .001.

Table S4

Results of Confirmatory Factor Analysis for the WHO-5

	**WHO-5**
Over the last two weeks, I have felt cheerful and in good spirit	1.000
Over the last two weeks, I have felt calm and relaxed	0.927
Over the last two weeks, I have felt active and vigorous	0.614
Over the last two weeks, I woke up feeling fresh and rested	0.645
Over the last two weeks, my daily life has been filled with things that interest me	0.962
CFI	.973
TLI	.941
RMSEA, 90% CI	.082, [.017, .144]
SRMR	.033
X^2	11.686
df	5

X^2/df	2.337
Cronbachs's alpha	.810

Note. N = 201. Models estimated with robust standard errors (MLR). CFA = Confirmatory factor analysis; SCF = Scaling correction factor for MLR; CFI = Comparative fit index; CI = Confidence interval; df = Degrees of freedom; RMSEA = Root mean square approximation; SRMR = Standard root means square residual; TLI = Tucker-Lewis Index.
* p < .05, ** p < .01, *** p < .001.

Table S5

Results of Confirmatory Factor Analysis for the German Flourishing Scale (FS-D)

	**FS-D**
I lead a purposeful and meaningful life.	1.000
My social relationships are supportive and rewarding.	0.700
I am engaged and interested in my daily activities.	0.945
I actively contribute to the happiness and well-being of others.	0.592
I am competent and capable in the activities that are important to me.	0.980
I am a good person and live a good life.	0.737
I am optimistic about my future.	0.979
People respect me.	0.548
CFI	.964
TLI	.950
RMSEA, 90% CI	.070, [.037, .102]
SRMR	.040
X^2	39.414
df	20
X^2/df	1.971
Cronbachs's alpha	.87

Note. N = 201. Models estimated with robust standard errors (MLR). CFA = Confirmatory factor analysis; SCF = Scaling correction factor for MLR; CFI = Comparative fit index; CI = Confidence interval; df = Degrees of freedom; RMSEA = Root mean square approximation; SRMR = Standard root means square residual; TLI = Tucker-Lewis Index.
* p < .05, ** p < .01, *** p < .001.

Table S6

Response Frequencies for Enacted, Internalized, and Anticipated Stigma Items of the Long-COVID Stigma Scale (N = 201)

Response options	0 (never)	1 (rarely)	2 (sometimes)	3 (often)	4 (always)
**Enacted stigma items**					
Because of my illness, some people seemed uncomfortable with me *[Wegen meiner Krankheit fühlten sich einige Leute nicht wohl mit mir.]*	25.4	26.4	38.8	9.5	
Because of my illness, some people were unkind to me *[Wegen meiner Krankheit waren einige Leute unfreundlich zu mir.]*	27.4	32.3	26.9	12.4	1.0
People I care about stopped contacting me after learning I have Long Covid *[Menschen, die mir wichtig sind, haben aufgehört, mich zu kontaktieren, nachdem sie erfahren haben, dass ich Long Covid habe.]*	46.8	26.4	18.4	7.5	1.0
People have acted as if I am dishonest since I have had Long Covid *[Seit ich Long Covid habe, verhalten sich die Menschen, als ob ich unehrlich wäre.]*	33.3	32.3	19.9	12.9	1.5
I have been treated with less respect than other people are because of Long Covid *[Ich bin wegen Long Covid mit weniger Respekt behandelt worden als andere Menschen]*	28.4	26.4	31.3	12.9	1.0
**Internalised stigma items**					
I have felt embarrassed about my illness *[Ich habe mich wegen meiner Krankheit beschämt gefühlt.]*	33.8	19.9	25.9	17.4	3.0
I have felt embarrassed because of my physical limitations *[Ich habe mich wegen meiner körperlichen Einschränkungen beschämt gefühlt.]*	18.9	22.4	26.9	28.4	3.5
I feel that I have been tainted by Long Covid and am of less value than others because of it *[Ich habe das Gefühl, dass ich durch Long Covid verdorben wurde und deshalb weniger wert bin als andere.]*	55.7	17.9	19.9	4.5	2.0
I have felt like I am very different from	6.5	11.9	30.3	36.8	14.4

other people on account of Long Covid *[Ich habe das Gefühl, dass ich mich wegen Long Covid sehr von anderen Menschen unterscheide.]*					
**Anticipated stigma items**					
Many people tend to think Long Covid isn’t a real illness *[Viele Menschen neigen dazu, zu denken, dass Long Covid keine echte Krankheit ist.]*	2.5	10.9	33.3	47.3	6.0
I feel that some people assume that having Long Covid is a sign of personal weakness *[Ich habe das Gefühl, dass manche Menschen davon ausgehen, dass Long Covid ein Zeichen von persönlicher Schwäche ist.]*	9.5	19.9	34.8	33.3	2.5
I worry that people with Long Covid lose their jobs when their employers find out *[Ich befürchte, dass Menschen mit Long Covid ihren Arbeitsplatz verlieren, wenn ihre Arbeitgeber es herausfinden.]*	14.5	21.0	40.5	22.0	2.0
I worry that people may judge me negatively when they learn I have Long Covid *[Ich befürchte, dass Menschen mich negativ beurteilen könnten, wenn sie erfahren, dass ich Long Covid habe.]*	26.4	21.4	37.3	12.9	2.0

Note. German items appear in *italic* below each English item.

Figure S1

Distribution of Responses in Percentages of Long-COVID Stigma Scale Items

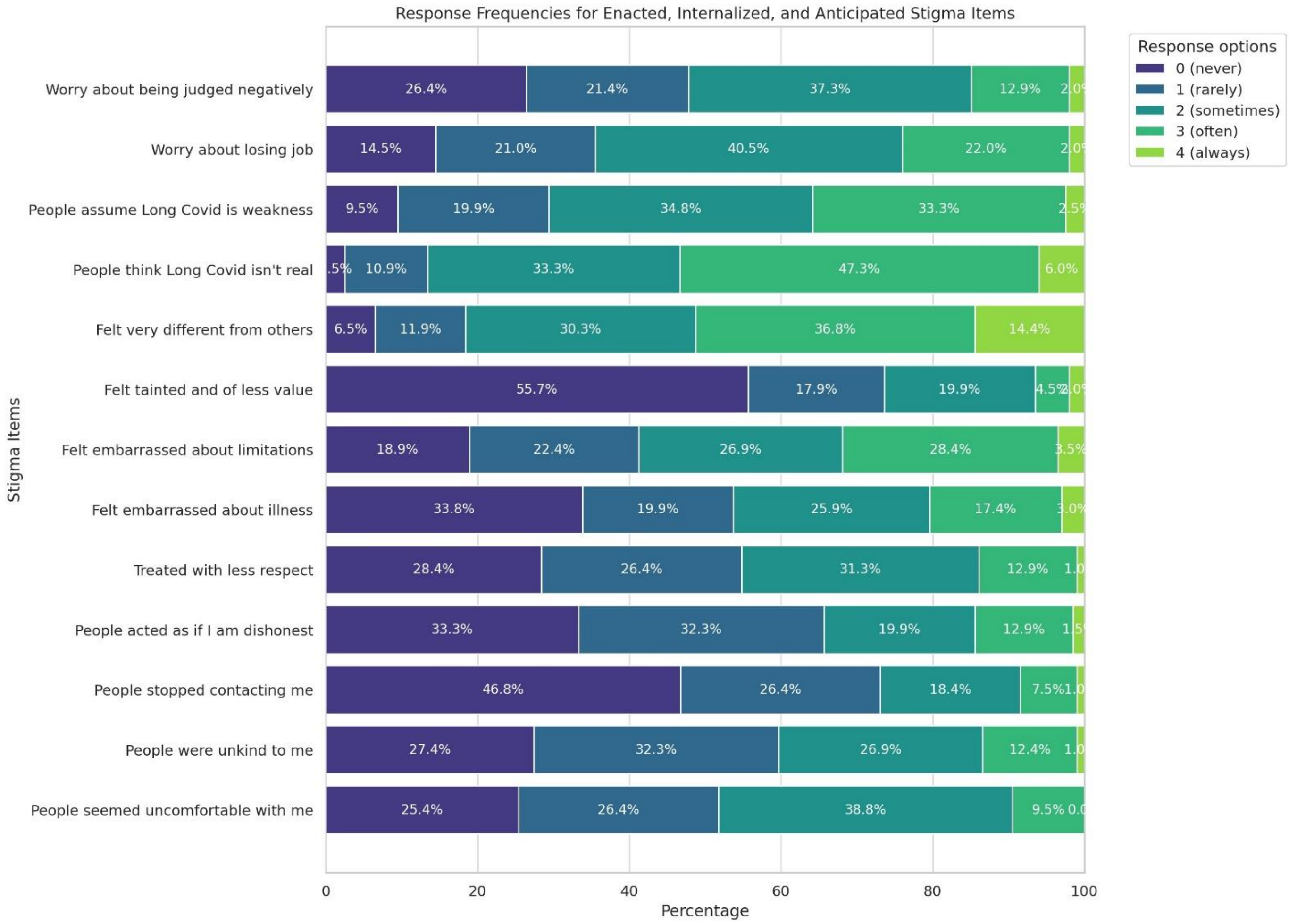

Note. N = 201. Items were shortened/summarized to fit the plot.

Table S7

Tests of Normality (N = 201)

	Shapiro-Wilk-Test				
	Statistic	Df	p value	Skewness	Kurthosis
**Long Covid Stigma Scale**					
Enacted stigma	0.963	201	< .001	0.451	2.573
Internalized stigma	0.976	201	.001	0.229	2.555
Anticipated stigma	0.976	200	.002	-0.276	2.805
**Self-Compassion-Scale**					
Self-compsssion	0.992	199	.299	-0.240	2.690
Self-coldness	0.981	200	.007	0.204	2.328
**WHO-5**	0.968	200	< .001	0.444	2.589
**FS-D**	0.978	198	.003	-0.467	2.881
**Long-COVID burden**	0.922	201	< .001	-0.839	4.241

Note. N = 201.

Table S8

Specific Indirect Effects in the Path Analysis for the Mediation Role of Self-Compassion and Self-Coldness in the Relationship Between Long-COVID Stigma and Well-Being, Controlling for Gender, Age, Income, Long-COVID Burdan, and Time Since Symptoms

**Path of Indirect Effect**	**B**	**SE**	**95% Bootstrapped CI**	**Adaptive / maladaptive role**
**Self-Compassion**				
Enacted stigma → PWB	0.013	0.021	[-0.011, 0.083]	
Internalized stigma → PWB	-0.046	0.036	[-0.188, 0.023]	
Anticipated stigma → PWB	-0.012	0.023	[-0.090, 0.014]	
Enacted stigma → Flourishing	0.026	0.032	[-0.019, 0.113]	
Internalized stigma → Flourishing	-0.091	0.045	[-0.198, -0.021]	Maladaptive
Anticipated stigma → Flourishing	-0.023	0.035	[-0.119, 0.029]	
**Self-Coldness**				
Enacted stigma → PWB	0.054	0.037	[0.001, 0.153]	Adaptive
Internalized stigma → PWB	-0.182	0.059	[-0.315, -0.080]	Maladaptive
Anticipated stigma → PWB	-0.069	0.041	[-0.173, -0.007]	Maladaptive
Enacted stigma → Flourishing	0.077	0.049	[0.001, 0.200]	Adaptive
Internalized stigma → Flourishing	-0.259	0.075	[-0.421, -0.130]	Maladaptive
Anticipated stigma → Flourishing	-0.098	0.055	[-0.236, -0.010]	Maladaptive

Note. *N* = 201. PWB = Psychological Well-Being (WHO-5). Controlling for gender (1 = female), age, income, long-COVID burden, and time since symptoms started (10 categories with equal frequencies).
